# Advanced oxidation protein products downregulate CYP1A2 and CYP3A4 expression and activity via the NF-κB-mediated signaling pathway in vitro and in vivo

**DOI:** 10.1038/s41374-021-00610-9

**Published:** 2021-05-24

**Authors:** Tianrong Xun, Zhufen Lin, Xiaokang Wang, Xia Zhan, Haixing Feng, Danna Gan, Xixiao Yang

**Affiliations:** 1 Department of Pharmacy, Shenzhen Hospital, Southern Medical University, Shenzhen, China; 2 Department of Pharmacy, Nanfang Hospital, Southern Medical University, Guangzhou, China

**Keywords:** Chronic kidney disease, Risk factors

## Abstract

Uremic toxin accumulation is one possible reason for alterations in hepatic drug metabolism in patients with chronic kidney disease (CKD). However, the types of uremic toxins and underlying mechanisms are poorly understood. In this study, we report the role of advanced oxidation protein products (AOPPs), a modified protein uremic toxin, in the downregulation of cytochromes P450 1A2 (CYP1A2) and P450 3A4 (CYP3A4) expression levels and activities. We found that AOPP accumulation in plasma in a rat CKD model was associated with decreased protein levels of CYP1A2 and CYP3A4. CYP1A2 and CYP3A4 metabolites (acetaminophen and 6β-hydroxytestosterone, respectively,) in liver microsomes were also significantly decreased. In human hepatocytes, AOPPs significantly decreased CYP1A2 and CYP3A4 protein levels in a dose- and time-dependent manner and downregulated their activities; however, bovine serum albumin (BSA), a synthetic precursor of AOPPs, had no effect on these parameters. The effect of AOPPs was associated with upregulation of p-IKKα/β, p-IκBα, p-NF-κB, and inflammatory cytokines protein levels and increases in p-IKKα/β/IKKα, p-IκBα/IκBα, and p-NF-κB/NF-κB phosphorylation ratios. Further, NF-kB pathway inhibitors BAY-117082 and PDTC abolished the downregulatory effects of AOPPs. These findings suggest that AOPPs downregulate CYP1A2 and CYP3A4 expression and activities by increasing inflammatory cytokine production and stimulating NF-κB-mediated signaling. Protein uremic toxins, such as AOPPs, may modify the nonrenal clearance of drugs in patients with CKD by influencing metabolic enzymes.

## Abstract (web summary)

Uremic toxin accumulation can alter hepatic drug metabolism. Advanced oxidation protein products significantly decrease the expression and activity of CYP1A2 and CYP3A4 via direct activation of the NF-κB pathway. Induction of nuclear translocation of NF-κB inhibits the transcription and translation of CYP1A2 and CYP3A4. The inflammatory cytokines IL-6 and/or TNF-α may mediate this process.

## Introduction

Patients with chronic kidney disease (CKD) require a mean of 14.2 concurrent drugs to manage their complications and associated comorbidities, and overdose or underdose errors account for 20.4% of all medical problems in these patients [1]. Dosing alterations for many renally cleared drugs in CKD patients have been well characterized; however, hepatic metabolism is also altered in CKD [2]. Drug pharmacokinetics are the result of the coordinated action of various drug-metabolizing enzymes, including cytochromes P450 (CYPs), and this coordinated action is highly altered in CKD [3]. These alterations are poorly understood, but numerous animal studies have indicated that CKD and decreased hepatic metabolism (including metabolism involving CYPs) are closely associated [4]. Moreover, CYPs activity is reduced in patients with CKD [5, 6].

CYP3A4 is the main CYP3A family member and is responsible for the metabolism of ~50% of clinically used drugs. Many of these drugs are used to treat CKD or its comorbidities [7, 8]. Alterations in the pharmacokinetics of CYP1A2-metabolized drugs have been reported in CKD [3]. Studies on uremic serum collected from patients with end-stage renal disease (ESRD) have indicated that ESRD decreases the protein expression and activities of the major xenobiotic-metabolizing CYP1A and CYP3A families in rat hepatocytes [9, 10]. Furthermore, intravenously administering a CYP3A4 substrate led to 6-fold higher systemic exposure in patients with ESRD compared to healthy controls [11]. Thus, the activities of CYP1A2 and CYP3A4 are decreased in CKD and are expected to have significant clinical implications, with altered drug pharmacokinetics and increased adverse drug events in patients with CKD. However, specific CKD-related factors that downregulate the activities of CYP1A2 and CYP3A4 have not been identified. Uremic toxins, which accumulate in the body in patients with CKD, may be involved [12, 13]. Nevertheless, the underlying molecular mechanism remains unknown.

AOPPs are a family of dityrosine-containing protein products that have been detected in the plasma of chronic uremic patients [14]. AOPPs are novel oxidative stress biomarkers and proinflammatory mediators and are related to oxidation-associated diseases [15]. AOPPs are high-affinity ligands for RAGE and CD36 and increase inflammatory cytokine (IL-6 and TNF-α) production and oxidant-dependent activation of the NF-κB signaling pathway in human chondrocytes [16, 17]. AOPPs have been studied extensively from their structural and pharmacokinetic properties to their clinical significance.

The NF-κB signaling pathway plays a key role in the regulation of the inflammatory cytokine production and NF-κB binds to CYP450 promoter regions to regulate CYP450 transcription and translation [18]. Activation of the NF-κB pathway is mediated by an increase in the phosphorylation of IKK and interaction with the inhibitory protein inhibitor κB (IκB) that binds to NF-κB and sequesters it in the cytoplasm. Upon stimulation with exogenous substances, IκB is phosphorylated by IκB kinases and then degraded by the proteasome. This prevents IκB from sequestering NF-κB in the cytoplasm and leads to NK-κB translocation to the nucleus and activation or silencing of its target genes [19, 20]. We hypothesized that AOPPs increase inflammatory cytokine production and activate the NF-κB signaling pathway, thereby regulating drug metabolism.

The objective of this study was to determine whether AOPPs can modify the expression of CYP1A2 and CYP3A4 and whether inflammatory cytokines and the NF-κB signaling pathway are involved in this process. To address this issue, we utilized a rat model of CKD to investigate the effect of AOPPs on CYP1A2 and CYP3A4 expression and activities in the liver. Thereafter, AOPPs-mediated downregulation of CYP1A2 and CYP3A4 was investigated in HepG2 and L-02 cells. Finally, we suggest a molecular mechanism by which AOPPs downregulate CYP1A2 and CYP3A4 expression and activity.

## Materials and methods

### Chemicals and reagents

BAY-117082, pyrrolidine dithiocarbamate (PDTC), 4’,6-diamidino-2-phenylindole (DAPI) and bovine serum albumin (BSA) were purchased from Sigma-Aldrich (St. Louis, MO, USA). Clorox was purchased from Macklin (Shanghai, China). Polyvinylidene fluoride (PVDF) membranes and a western blot detection system were purchased from Millipore (Darmstadt, Germany). Primary antibodies against CYP1A2 (DF3565), CYP3A4 (DF7001), histone H3 (AF0863), and GAPDH (AF7021) were purchased from Affinity Biosciences (California, USA). Primary antibodies against NF-κB (8242), p-NF-κB (3039), IKBα (4812), p-IKBα (5209), IKKα (2682), p-IKKα/β (2697), and horseradish peroxidase-conjugated goat anti-mouse (7076 S) and goat anti-rabbit (7074 S) IgG were purchased from Cell Signaling Technology (Beverly, MA, USA). Tris-buffered saline with Tween 20 (TBST), antibody dilution buffer, and a pNF-κB-luc reporter plasmid were purchased from Beyotime (Shanghai, China).

### Preparation of AOPPs

AOPPs were prepared in vitro as described previously [21]. Briefly, 20 mg/ml BSA solution was combined with 40 mmol/L Clorox at a molar ratio of 1:140 for 30 min at room temperature protected from light. The samples were then dialyzed overnight against phosphate-buffered saline (PBS) to remove the free Clorox. Endotoxin levels in AOPP solutions were determined using an Amebocyte lysate assay kit (T-125; Zhanjiang, China). BSA dissolved in PBS was used as control.

### Animal model

All animal studies were approved by the institutional Animal Experiment Committee of the Southern Medical University (Guangzhou, China). A total of 39 male Sprague–Dawley rats (160–200 g) were obtained from the Southern Medical University Animal Experiment Center (Guangzhou, China). The rats were treated according to a two-step procedure as described previously [22]. Briefly, the rats underwent five-sixths nephrectomy (5/6 nx; *n* = 21 (3 later excluded due to surgery-related death) or sham operation (sham; *n* = 18). Two weeks later, the 5/6 nx and sham rats were randomized into subgroups (groups 1–3; 5/6 nx, and sham groups 4–6; *n* = 6 per group). The rats in received intraperitoneal injections as follows once every other day for 8 weeks: (1) PBS (groups 1 and 4), (2) unmodified BSA (30 mg/kg, groups 2 and 5), and (3) AOPPs (30 mg/kg, groups 3 and 6). After 8 weeks, the rats were anesthetized with 3% pentobarbital sodium (v/v), and plasma samples were collected. The left kidney, the liver and the intestine were collected in liquid nitrogen.

### AOPPs concentration determination

The concentration of AOPPs in the rat plasma was determined by spectral analysis as described previously [14]. Aliquots of 200 μl of chloramine-T for calibration (0–100 μmol), 200 μl of serum samples (diluted 1:5 in PBS), and 200 μl of PBS as a blank control were placed in a 96-well plate. Subsequently, 10 μl of 1.16 M potassium iodide and 20 μl of acetic acid were added to each well. The absorbance of the reaction system at 340 nm was immediately measured. The AOPPs concentration was expressed as micromoles of chloramine-T equivalents per liter. The AOPPs concentration divided by the albumin level was regarded as the AOPPs level.

### Western blotting

Cultured HepG2 and L-02 cells or frozen rat liver tissue samples were lysed with radioimmunoprecipitation assay (RIPA) lysis buffer containing protease inhibitors. The cytoplasmic and nuclear proteins were extracted with a nuclear and cytoplasmic protein extraction kit (KGP150; KeyGEN BioTECH, Jiangsu, China) according to the manufacturer’s instructions. Equal amounts of protein were separated by sodium dodecyl sulfate polyacrylamide gel electrophoresis (PAGE) using 10% acrylamide gels and then transferred to polyvinylidene fluoride (PVDF) membranes. The membranes were incubated with the following primary antibodies overnight at 4 °C: CYP1A2 (1:500), CYP3A4 (1:500), GAPDH (1:1000), NF-κB (1:1000), p-NF-κB (1:1000), IKBα (1:1000), p-IKBα (1:1000), IKKα (1:1000), p-IKKα/β (1:1000), and histone H3 (1:1000; used as a reference antibody for nuclear proteins). The membranes were washed with TBST and incubated with horseradish peroxidase (HRP)-conjugated goat anti-rabbit IgG (1:3,000) or goat anti-mouse IgG (1:3000) for 1 h at room temperature. The signals were visualized using enhanced chemiluminescence (ECL) detection, and a densitometric analysis was performed using imaging software. The protein levels were normalized to GAPDH or histone H3.

### Hepatic microsome isolation

Hepatic microsomes were isolated by standard differential centrifugation procedures as previously described with a minor modification [23]. In brief, liver tissue was perfused with prechilled buffer comprising 1.5 mM EDTA, 1 mM DTT, 8 mM KH_2_PO_4_, 0.28 mM PMSF, and 5.6 mM Na_2_HPO_4_. Thereafter, the collected tissue was shredded and homogenized in 50 mM buffer (pH = 7.4) comprising 1 mM EDTA, 0.28 mM PMSF, and 250 mM sucrose. The mixture was centrifuged at 12,000 × g and 4 °C for 15 min and the sediment was discarded. Next, the supernatant was centrifuged at 35,000 × g and 4 °C for 1 h. The microsomes were resuspended in 250 mM sucrose and the mixture was immediately stored at −80 °C. The concentration of hepatic microsomes was determined using a bicinchoninic acid (BCA) protein assay kit (KGSK4051; KeyGEN BioTECH, Jiangsu, China).

### Metabolism of CYP1A2 and CYP3A4 substrates

The metabolic activities of CYP1A2 and CYP3A4 in hepatic microsomes was determined using specific probe substrates. Phenacetin was used as a probe for CYP1A2 [24] and testosterone was used as a probe for CYP3A4 [25]. Briefly, microsomes (0.4 mg/ml) were mixed on ice with 50 mM buffer (pH = 7.4) comprising 1.5 mM EDTA, 1 mM DTT, 8 mM KH_2_PO_4_, 0.28 mM PMSF, and 5.6 mM Na_2_HPO_4_, substrate, and a NADPH-generating system in a final incubation volume of 250 μl. The mixture was incubated in a shaking water bath at 37 °C for 30 min. The reaction was terminated by adding 200 μl of cold methanol and the mixture was centrifuged at 15,000 × g for 30 min. The supernatant was collected in a glass injection bottle.

Acetaminophen and 6β-hydroxytestosterone was assessed by high-performance liquid chromatography (HPLC) system (Shimadzu, Japan) with an Eclipse Plus C18 column (5 μm, 4.6 × 250 mm; Agilent). A sample volume of 20 μl was injected into the column. The sample rack and column were maintained at 30 °C and the flow rate was maintained at 0.75 ml/min. Mobile phase A was HPLC-grade water with 0.1% (v/v) formic acid, and mobile phase B was 100% acetonitrile. An ultraviolet detector was used to detect acetaminophen (257 nm) and 6β-hydroxytestosterone (245 nm). The quantification of acetaminophen and 6β-hydroxytestosterone was based on the peak area.

### Immunofluorescence staining

Immunofluorescence staining was performed using an NF-κB activation and nuclear translocation assay kit (SN368; Beyotime) according to the manufacturer’s instructions with minor modifications. In brief, fixed cells were blocked with a sealing fluid for 1 h at room temperature. The cells were then incubated with rabbit anti-NF-κB antibody overnight at 4 °C. After incubation with Cy5-conjugated anti-rabbit IgG for 1 h, the nuclei were stained with DAPI (1 µg/ml). Fluorescent signals were captured with an FluoView FV10i self-contained confocal laser scanning microscope (Olympus, USA).

### Transient transfection and reporter gene assay

A total of 0.5 μg of pNF-κB-luc reporter plasmid or the equivalent amount of a control plasmid (D2206; Beyotime) was transfected into cells using Lipofectamine 3000 reagent (1741089; Life Technologies, Waltham, MA, USA) according to the manufacturer’s protocol. After transfection for 24 h, the cells were treated with AOPPs or without AOPPs (CON). Thereafter, cell lysis was carried out using reporter lysis buffer (RG055M; Beyotime). The cell extract was mixed with a luciferase substrate and the luciferase activity was immediately determined using a luminometer microplate reader (Spectrometer MD5; XYZ, USA).

### Enzyme linked immunosorbent assay (ELISA)

After coculture with AOPPs, the concentrations of proinflammatory cytokines were measured in supernatants prepared using plasma or macrophages from the rats subjected to various treatments. The levels of TNF-α and IL-6 in the supernatants were detected using ELISA kits (RA20035 and RA20607; Bio-Swamp, China) according to the manufacturer’s instructions.

### Statistical analysis

The data are expressed as mean ± standard deviation (SD). A Michaelis–Menten model was used to fit the data regarding phenacetin and testosterone metabolite formation. The statistical analyses were performed in GraphPad Prism software (GraphPad 5.0 Software, USA). The data were analyzed using Student’s *t* test or one-way analysis of variance (ANOVA), with the least significant difference test being used as the post-hoc test. The data were also analyzed using three-factor ANOVA. Differences were considered statistically significant at *p* < 0.05.

## Results

### AOPPs administration increased AOPPs accumulation in plasma

**Fig. 1:**
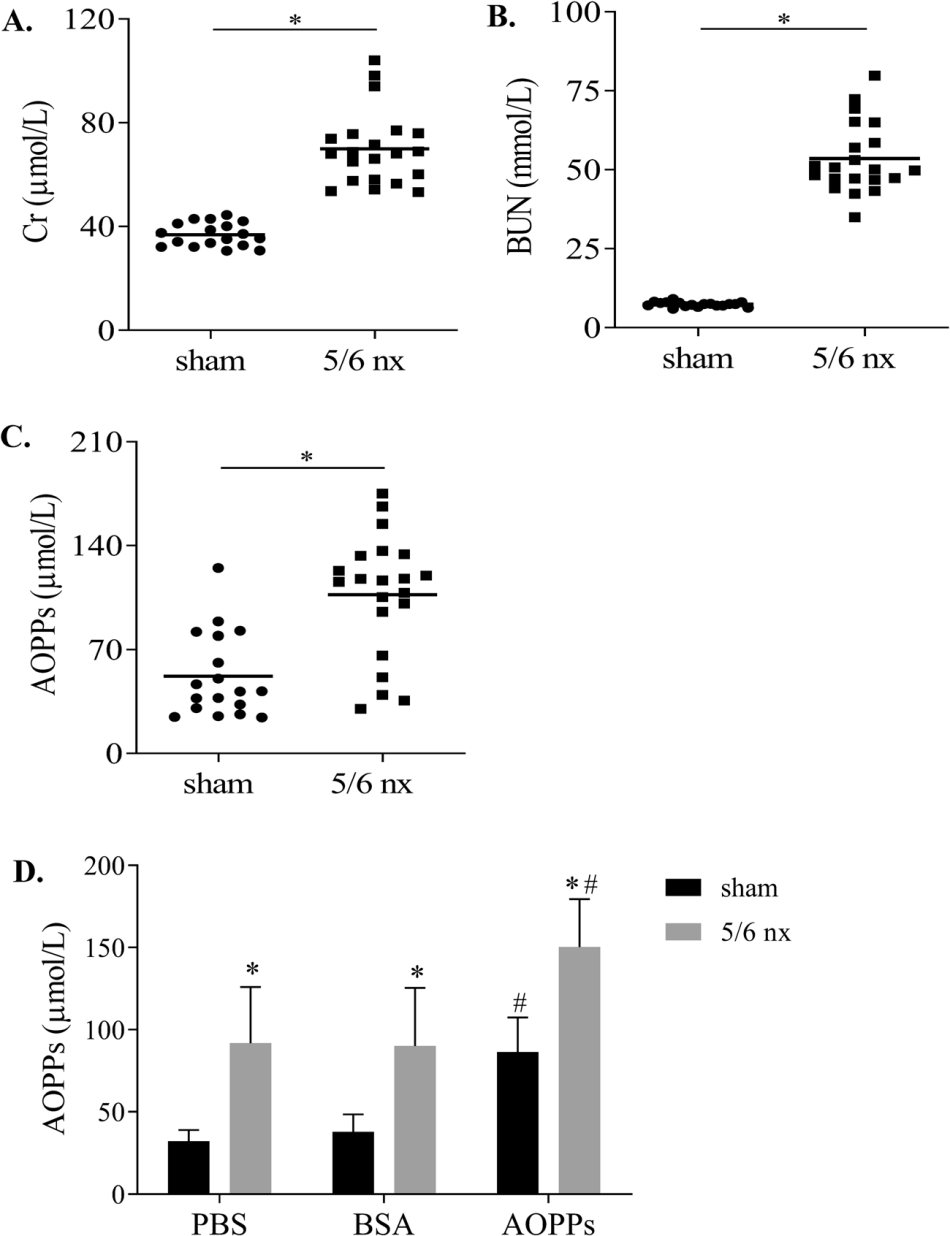
AOPPs administration increased AOPPs accumulation in the plasma. Kidney function of 5/6 nx rats was assessed based on Cr (**A**) and BUN (**B**), which indicated reduced renal function. Plasma AOPPs concentration was increased in 5/6 nx rats (**C**). After AOPPs administration, the plasma AOPPs concentration in the sham and 5/6 nx rats increased (**D**). The results are shown as a scatter plot and each point represents one rat. *p < 0.05 compared with the sham group; ^#^*p* < 0.05 compared with the PBS group.

In the CKD model, renal function parameters, including plasma creatinine (Cr) (Fig. 1A), blood urea nitrogen (BUN) (Fig. 1B) and AOPPs (Fig. 1C), were increased by 1.9-fold, 7.3-fold, and 2.0-fold compared with those in the sham group. After AOPPs administration, AOPPs were increased in the sham and 5/6 nx groups (Fig. 1D), 2.6-fold and 1.6-fold compared with that in the PBS group. These data suggest that AOPPs administration increases AOPPs accumulation in the plasma.

### AOPPs downregulated CYP1A2 and CYP3A4 protein levels in the intestine, kidney, and liver

**Fig. 2:**
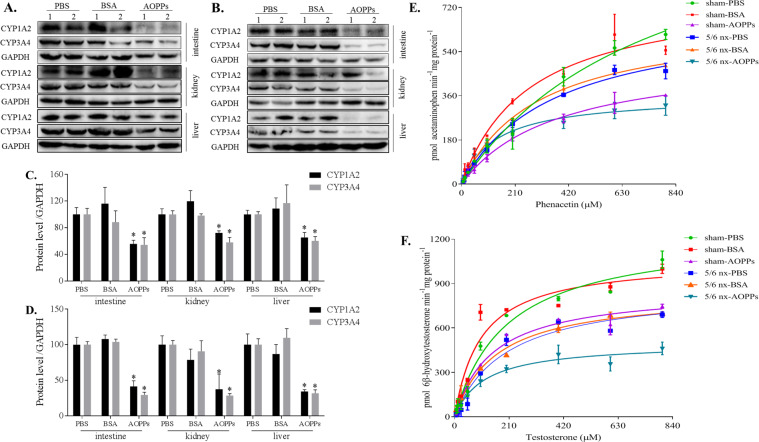
AOPPs downregulated the protein expression and activities of CYP1A2 and CYP3A4 in vivo. Total protein was extracted from the intestine, kidney, and liver in the sham (**A**) and 5/6 nx groups (**B**), and the protein expression of CYP1A2 and CYP3A4 in a whole-cell lysate was evaluated by western blotting. Proteins expression levels were quantified by ImageJ software (**C**, **D**). Each experiment was performed with a different isolate. Michaelis–Menten plots of acetaminophen (**E**) and 6β-hydroxytestosterone (**F**) were constructed after incubation of liver microsomes (extracted from the liver tissues in the sham and 5/6 nx groups) with NADPH and various concentrations of phenacetin or testosterone, respectively, which were used to evaluate the activities of CYP1A2 and CYP3A4, respectively. Each data point represents the mean of three replicates and the error bars represent standard error of the mean (*n* = 3). Data are presented as mean ± SD; *p < 0.05 compared with the PBS group. Data were normalized to GAPDH.

In the sham group, treatment with AOPPs reduced CYP1A2 protein levels in the intestine, kidney, and liver by 44%, 28%, and 35%; CYP3A4 protein levels in the intestine, kidney, and liver were reduced by 46%, 42%, and 40%, respectively (Fig. 2A). In the 5/6 nx group treated with AOPPs, CYP1A2 protein levels in the intestine, kidney, and liver were reduced by 59%, 63%, and 66% and CYP3A4 protein levels were reduced by 71%, 71%, and 68%, respectively, compared with those in the PBS or BSA groups (Fig. 2B).

### AOPPs downregulated the activities of CYP1A2 and CYP3A4 in the liver

The activities of CYP1A2 (Fig. 2E) and CYP3A4 (Fig. 2F) in the liver microsomes were evaluated using phenacetin and testosterone, respectively. After treatment with AOPPs, the V_max_ values for acetaminophen in the sham and 5/6 nx groups were reduced by 57% and 49% and the K_m_ values were reduced by 52% and 64%, respectively. The values of V_max_ for 6β-hydroxytestosterone in the AOPP-treated sham and 5/6 nx rats were reduced by 31% and 43%, respectively; the values of K_m_ were reduced by 27% and 42%, respectively, compared with those in the PBS or BSA groups.

### AOPPs downregulated CYP1A2 and CYP3A4 protein levels in HepG2 and L-02 cells

**Fig. 3:**
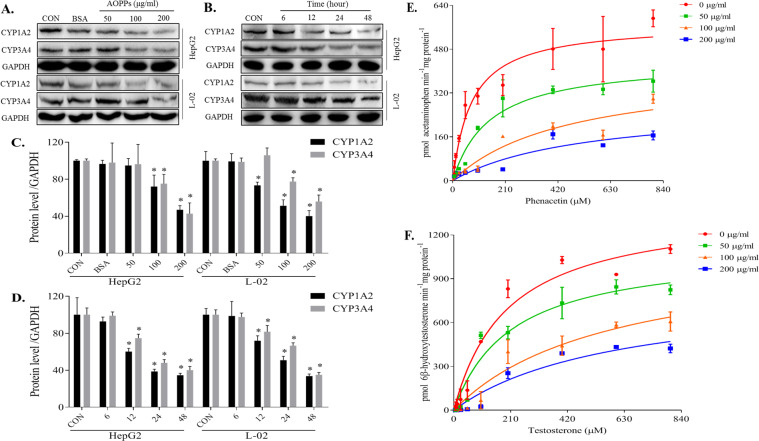
AOPPs downregulated the protein expression and activities of CYP1A2 and CYP3A4 in vitro. HepG2 and L-02 cells were treated with the control medium (CON), BSA, or the indicated AOPPs concentration. AOPPs treatment decreased the protein expression of CYP1A2 and CYP3A4 in a dose- (**A**) and time-dependent manner (**B**). Protein expression levels were quantified by ImageJ software (**C**, **D**). Each experiment was performed with a different isolate. Microsomes were treated with indicated concentration of AOPPs and the substrate. AOPPs dose-dependently decreased the activities of CYP1A2 (**E**) and CYP3A4 (**F**). Each data point represents the mean of three replicates and the error bars represent standard error of the mean (*n* = 3). Data are presented as mean ± SD; *p < 0.05 compared with the CON group. Data were normalized to GAPDH.

CYP1A2 and CYP3A4 protein levels in HepG2 and L-02 cells were gradually decreased concomitant to an increase in the AOPPs concentrations (Fig. 3A). After treatment with 100 µg/ml AOPPs, CYP1A2 protein levels in HepG2 and L-02 cells were reduced by 28% and 49% and the CYP3A4 levels were reduced by 25% and 23%, respectively. After treatment with 200 µg/ml AOPP, CYP1A2 protein levels in HepG2 and L-02 cells were reduced by 53% and 60% and the CYP3A4 levels were reduced by 57% and 44%, respectively. CYP1A2 and CYP3A4 protein levels in HepG2 and L-02 cells were gradually decreased over time (Fig. 3B). After treatment with 200 µg/ml AOPPs for 24 h, CYP1A2 protein levels in HepG2 and L-02 cells were reduced by 61% and 49% and the CYP3A4 levels were reduced by 52% and 33%, respectively. In summary, CYP1A2 and CYP3A4 levels were lower when the concentrations of AOPPs exceeded 100 μg/ml or when the incubation time reached 24 h.

### AOPPs downregulated CYP1A2 and CYP3A4 activities in the liver microsomes

AOPPs inhibited the production of acetaminophen (Fig. 3E) and 6β-hydroxytestosterone (Fig. 3F) in the rat liver microsomes in a dose-dependent manner. After treatment with 100 µg/ml AOPPs, the V_max_ values for acetaminophen and 6β-hydroxytestosterone in the microsomes were reduced by 29% and 21%; however, the K_m_ values were increased by 6.1-fold and 2.9-fold, respectively. After 200 µg/ml AOPP treatment, the V_max_ values were reduced by 52% and 41%; however, the K_m_ values were increased by 6.8-fold and 3.0-fold, respectively.

### AOPPs activated the IKK/IκB/NF-κB pathway in vitro and in vivo

**Fig. 4:**
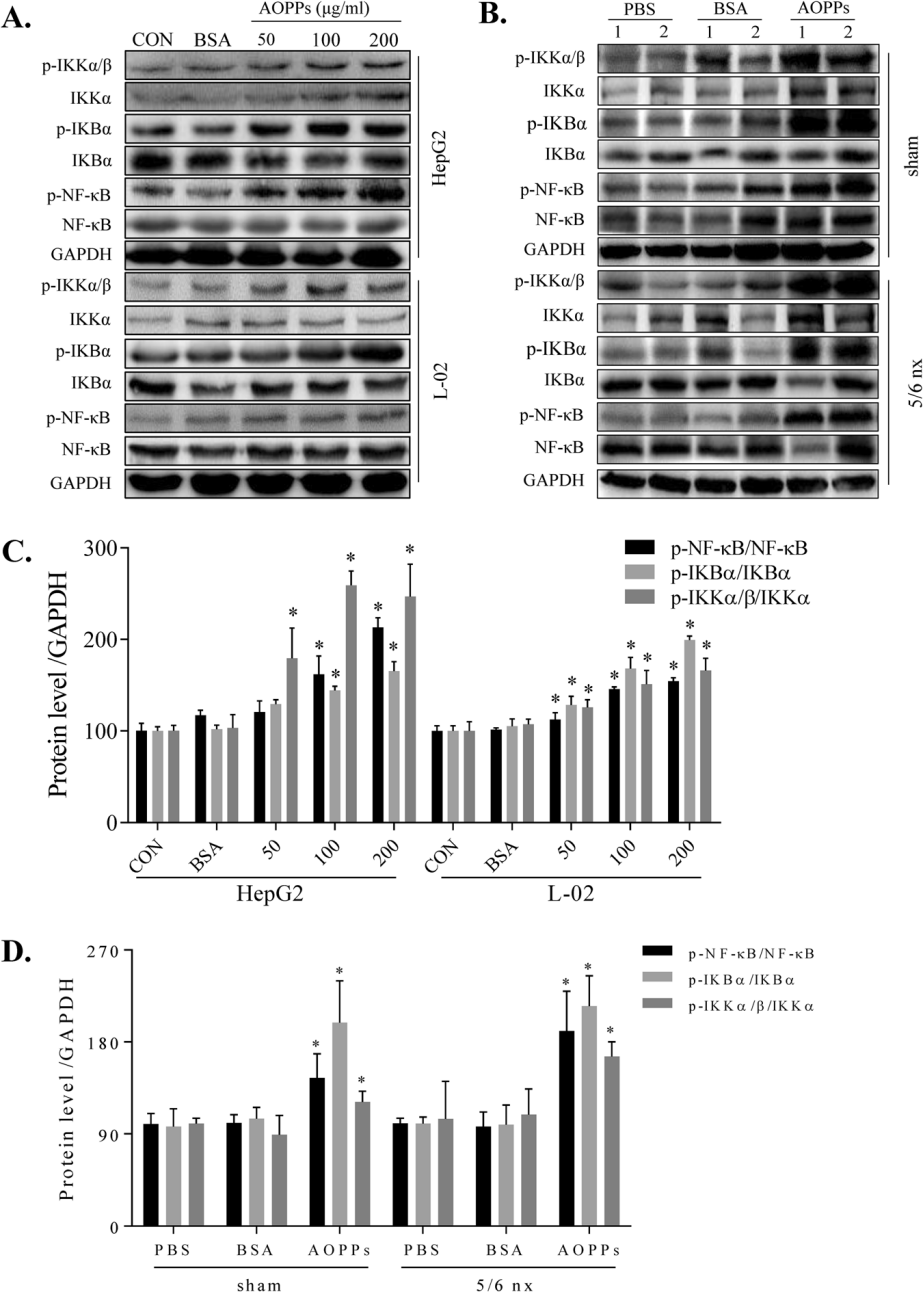
AOPPs activated the IKK/IκB/NF-κB pathway in vitro and in vivo. AOPPs increased the expression of p-NF-κB, p-IKBα, and p-IKKα/β in the total protein fraction of HepG2 and L-02 cells (**A**). AOPPs increased the expression of p-NF-κB, p-IKBα, and p-IKKα/β in the liver tissues of the sham and 5/6 nx groups (**B**). Protein expression levels were quantified by ImageJ software (**C**, **D**). Each experiment was performed with a different isolate. Data are presented as mean ± SD; *p < 0.05 compared with the CON or PBS groups. Data were normalized to GAPDH.

To investigate the mechanism responsible for the decrease in CYP1A2 and CYP3A4, we investigated the links between IKK/IκB signaling and NF-κB activation. In HepG2 and L-02 cells (Fig. 4A), AOPPs significantly and dose-dependently induced IKK/IκB/NF-κB phosphorylation compared to the CON group. BSA had no effect compared to the CON group. We next examined the effect of AOPPs on the protein expression of IKK/IκB/NF-κB in the liver of rats. AOPPs significantly increased p-IKKα/β, p-IκBα, and p-NF-κB protein levels in the sham and 5/6 nx rats and significantly increased the phosphorylation ratio of p-IKKα/β/IKKα, p-IκBα/IκBα, and p-NF-κB/NF-κB (Fig. 4B).

### AOPPs-induced nuclear translocation of NF-κB

**Fig. 5:**
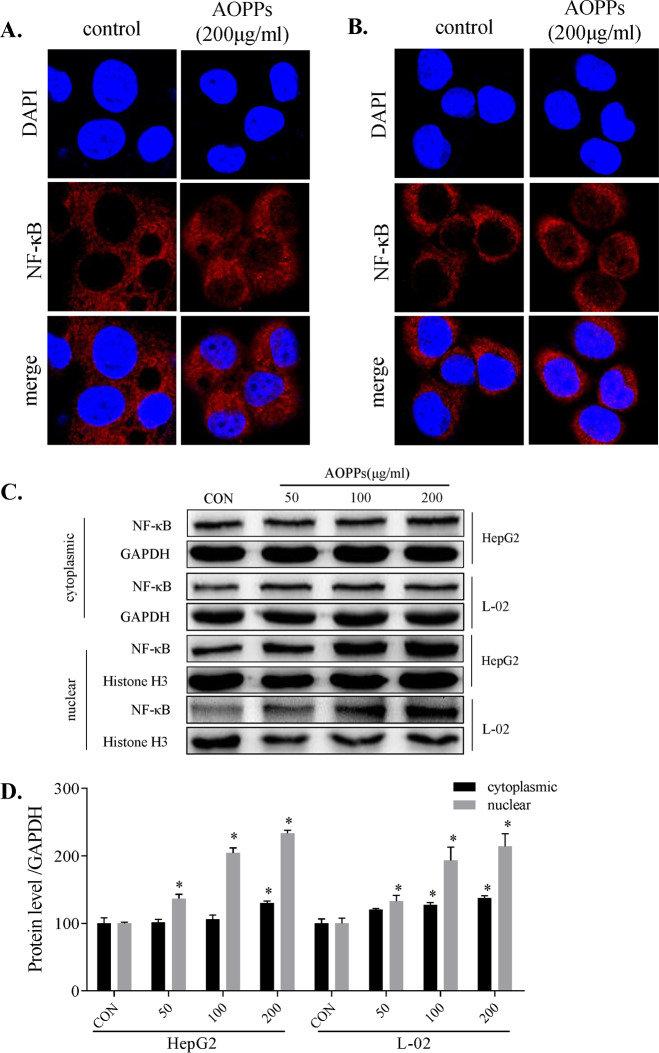
AOPPs-induced nuclear translocation of NF-κB. AOPPs increased nuclear translocation of NF-κB detected in the nuclear protein by laser confocal microscopy (400×) in HepG2 (**A**) and L-02 cells (**B**). Cytoplasmic and nuclear protein expression levels of NF-κB were detected by western blotting (**C**). Protein expression levels were quantified by ImageJ software (**D**). Each experiment was performed with a different isolate. Data are presented as mean ± SD; *p < 0.05 compared with the CON group. Data were normalized to GAPDH.

We assayed the nuclear translocation of the NF-κB protein induced by AOPPs using a confocal microscope. NF-κB protein in the nucleus was increased in HepG2 (Fig. 5A) and L-02 (Fig. 5B) cells, with almost no increase in the control group. NF-κB protein was detected in the cytoplasmic and nuclear protein fractions of HepG2 and L-02 cells, and NF-κB protein levels were slightly increased in the cytoplasm and significantly increased in the nucleus in a dose-dependent manner (Fig. 5C).

### AOPPs downregulated CYP1A2 and CYP3A4 expression via the NF-κB pathway

**Fig. 6:**
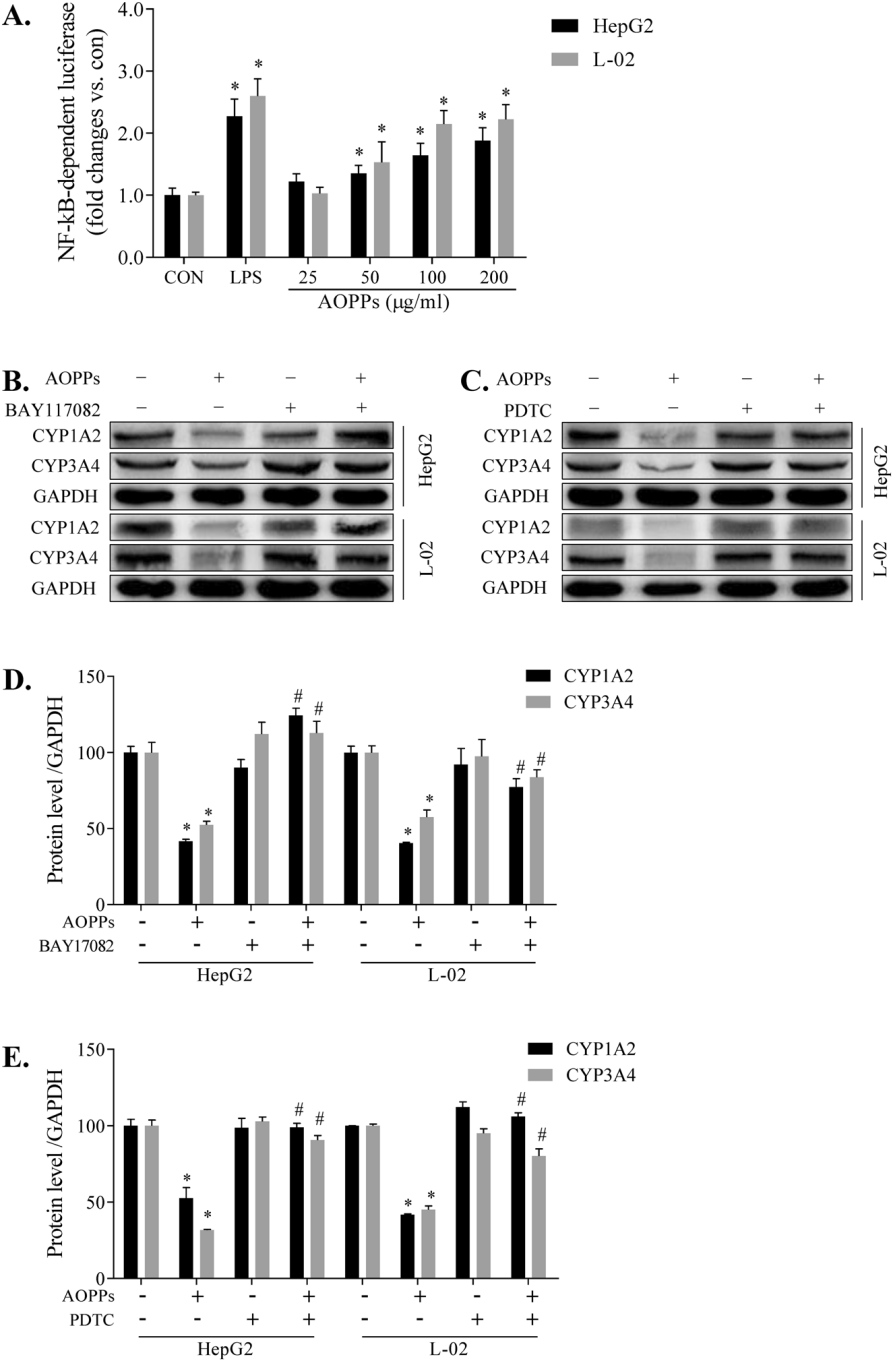
AOPPs downregulated CYP1A2 and CYP3A4 expression via the NF-κB pathway. NF-κB-dependent firefly luciferase reporter gene was transfected into HepG2 or L-02 cells, and the reporter gene expression (**A**). HepG2 or L-02 cells were treated with AOPPs (200 µg/ml) for 48 h and cocultured with BAY-117082 (**B**) and PDTC (**C**) to restore the downregulated expression levels of CYP1A2 and CYP3A4. Protein expression levels of CYP1A2 and CYP3A4 were quantified by ImageJ software (**D**, **E**). Each experiment was performed with a different isolate. Data are presented as mean ± SD; *p < 0.05 compared with the CON group; ^#^*p* < 0.05 compared with the group without BAY-117082 or PDTC. Data were normalized to GAPDH.

To gain an insight into NF-κB activation, we examined the effects of AOPPs on a luciferase reporter construct containing the functional gene followed by the coding sequence of the firefly luciferase gene. Similar to lipopolysaccharide, AOPPs can dose-dependently induce NF-κB pathway activation in HepG2 and L-02 cells compared with the CON groups of the corresponding cells, thus increasing the transcription and translation of the luciferase reporter gene (Fig. 6A). Inhibitors of the NF-kB pathway, 5 μM BAY-117082 (Fig. 6B) and 25 μM PDTC (Fig. 6C), effectively ameliorated the decrease in the CYP1A2 and CYP3A4 protein expression levels induced by AOPPs. The protein expression levels in the BAY-117082 (Fig. 6D) and PDTC (Fig. 6E) groups were quantified by the ImageJ software. These data suggest that AOPPs downregulate CYP1A2 and CYP3A4 expression via the NF-κB pathway.

### AOPPs downregulated CYP1A2 and CYP3A4 expression via the inflammatory cytokine pathway

**Fig. 7:**
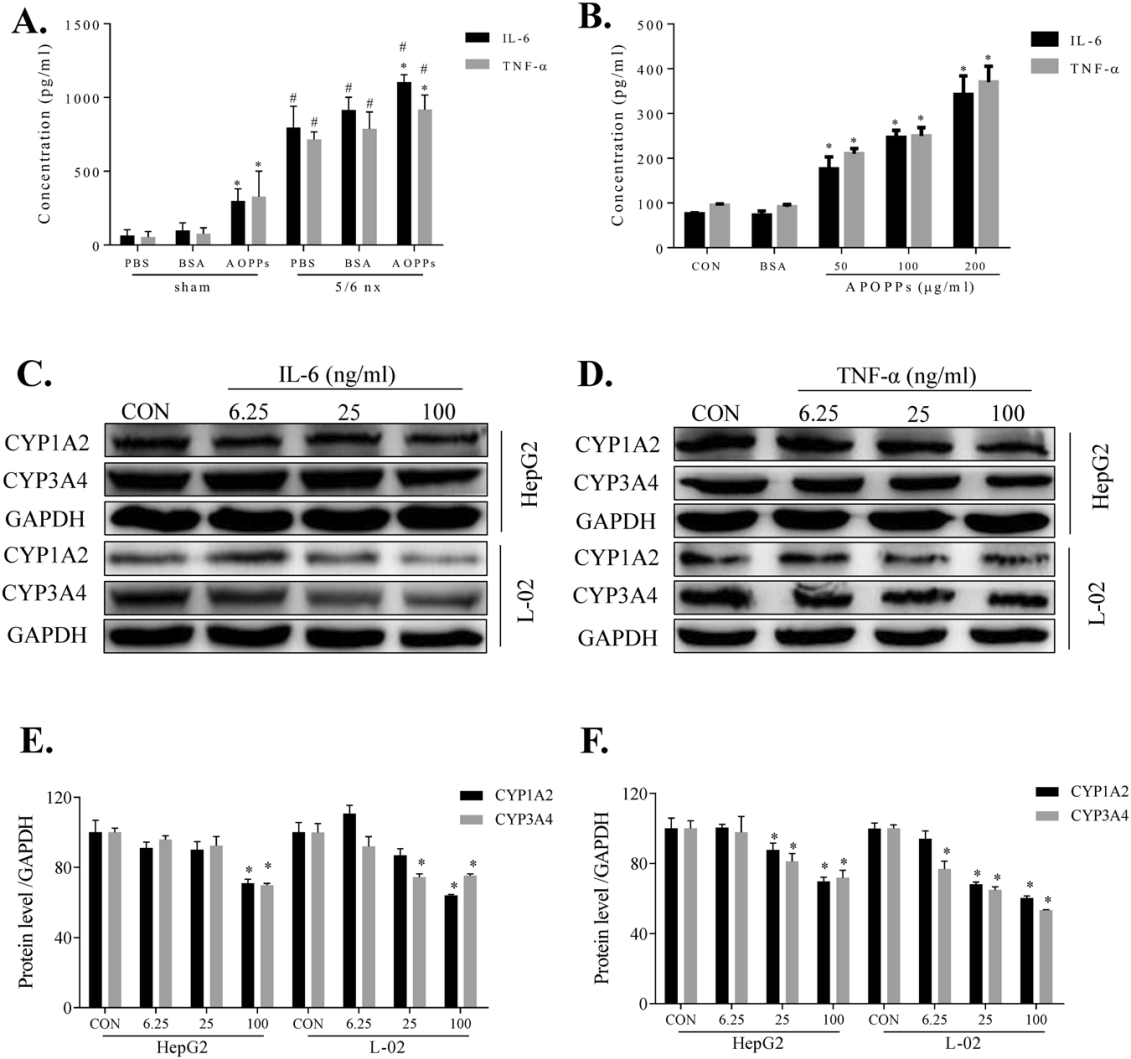
AOPPs downregulated CYP1A2 and CYP3A4 expression via the inflammatory cytokine pathway. AOPPs increased the plasma concentrations of IL-6 and TNF-α in the sham and 5/6 nx groups (**A**). AOPPs increased the IL-6 and TNF-α concentrations in rat macrophages (**B**). HepG2 and L-02 cells treated with various concentrations of IL-6 (**C**) or TNF-α (**D**) for 48 h exhibited dose-dependent decreases in the protein expression levels of CYP1A2 and CYP3A4, as evaluated by western blotting. Protein expression levels of CYP1A2 and CYP3A4 levels were quantified by ImageJ software (**E**, **F**). Each experiment was performed with a different isolate. Data are presented as mean ± SD; *p < 0.05 compared with the CON group, ^#^*p* < 0.05 compared with the sham group. Data were normalized to GAPDH.

Initially, the effect of AOPPs administration on the expression of TNF-α and IL-6 in the rat plasma and macrophages was examined by ELISA. After AOPPs administration, the concentrations of TNF-α in the plasma of the sham and 5/6 nx rats were increased by 6.0-fold and 1.3-fold and the levels of IL-6 were increased by 4.6-fold and 1.4-fold, respectively (Fig. 7A). In rat macrophages, AOPPs increased the levels of IL-6 and TNF-αin a dose-dependent manner with no effect of BSA. The levels were significantly higher than that detected in the CON group (Fig. 7B). Next, we found that IL-6 treatment caused a dose-dependent decrease in the protein expression of CYP1A2 and CYP3A4 in HepG2 and L-02 cells (Fig. 7C), as did TNF-α treatment (Fig. 7D). The protein expression levels in the IL-6 (Fig. 7E) and TNF-α (Fig. 7F) groups were quantified by the ImageJ software. The data suggest that TNF-α and IL-6 can directly influence the expression of CYP1A2 and CYP3A4 proteins.

## Discussion

This study demonstrates that AOPPs mediate the downregulation of CYP1A2 and CYP3A4 in vitro and in vivo. An exogenous increase in the AOPP plasma concentration significantly increased TNF-α and IL-6 release and reduced the expression and metabolic activities of CYP1A2 and CYP3A4 proteins in the liver. In human hepatocytes, purified AOPPs significantly reduced the protein expression of CYP1A2 and CYP3A4 by activating the relevant proteins in the NF-κB pathway. BAY-117082 and PDTC, inhibitors of the NF-kB pathway, significantly reversed the effect of AOPPs.

A number of studies have shown that CKD alters renal and nonrenal drug elimination; the accumulated uremic toxins and inflammatory cytokines may modulate metabolic enzymes either directly or by inhibiting protein expression [26]. New techniques to remove small-molecule or protein-bound uremic toxins from patients with CKD can still leave these patients with altered drug pharmacokinetics in response to certain drugs, and the pharmacokinetic parameters are difficult to assess [6]. To explore the effects of AOPPs in CKD, we used the 5/6 nx CKD model, in which the residual nephrons are characterized by elevated perfusion, filtration, and pressure, eventually leading to CKD. Markers of CKD are increased in this model by 8 weeks, such as serum creatinine, BUN and AOPPs (Fig. 1A–C). Elevated perfusion and filtration increase the effects of uremic toxins on the liver. However, the 5/6 nx model can limit liver function changes caused by CKD, unlike other CKD models (e.g., the adenine model).

AOPPs, a new class of renal pathogenic mediators of CKD progression that gradually increase in the blood with CKD progression, are dityrosine-containing crosslinking protein products formed during oxidative stress by reactions of plasma proteins with chlorinated oxidants [27]. AOPPs are primarily eliminated in the liver via the scavenger receptors in hepatic non-parenchymal cells and macrophages, and they are involved in liver disease progression via highly complex mechanisms [28]. Although several studies have investigated the profound effects of AOPPs regarding progressive liver function damage, there have only been a few studies on the potential mechanisms [29]. To the best of our knowledge, this study is the first to clarify that AOPPs mediate the downregulation of the expression and activities of CYP1A2 and CYP3A4 in the liver. The plasma AOPP concentration was significantly increased in the AOPPs-treated rats compared to the BSA- or PBS-treated rats, regardless of whether the rats were in the sham or 5/6 nx group (Fig. 1D). The increase plasma levels of significantly decreased the protein expression of the metabolic enzymes CYP1A2 and CYP3A4 in the liver (Fig. 2A, B), and the metabolism of the probe substrates was significantly decreased (Fig. 2E, F). Thus, AOPPs can reduce CYP1A2 and CYP3A4 function in the liver and thereby influence the normal physiological function of the liver.

To further explore the independence of the effect of AOPPs, we cocultured the HepG2 and L-02 cell lines with or without AOPPs to confirm the accuracy of our results. HepG2 cells are transformed human hepatocellular cells that maintain the basic characteristics of hepatocytes and exhibit CYP1A2 and CYP3A4 expression. L-02 cells are normal human hepatocytes that also exhibit CYP1A2 and CYP3A4 expression and can be used as control cells to compare to cancer cells [30, 31]. To confirm that the in vitro AOPPs experiments are relevant to the in vivo experiments, we performed dose- and time-dependent assays of AOPPs effects. In HepG2 or L-02 cells, AOPPs reduced the CYP1A2 and CYP3A4 protein expression in a dose- (Fig. 3A) and time-dependent (Fig. 3B) manner. Liver microsomes are a common model for specific detection of enzyme metabolism in vivo [12]. In this study, the effects of AOPPs on the metabolism of phenacetin (Fig. 3E) and testosterone (Fig. 3F) were explored. Phenacetin is a specific substrate of CYP1A2 and its main metabolite is acetaminophen [24]. Testosterone is a specific substrate of CYP3A4 and its main metabolite is 6β-hydroxytestosterone [25]. Each compound specifically responds to changes in the activity of the corresponding metabolic enzyme [13, 32]. The results of the CYP1A2 and CYP3A4 assays were similar to the in vivo data from rats. However, the marked increases in the K_m_ values in the liver microsome reaction system may be related to the non-specific binding of AOPPs.

AOPPs can induce reactive oxygen species production and activate the NF-kB pathway to produce a series of pathological processes [17, 33]. NF-κB plays an important role in mediating the suppression of drug-metabolizing enzymes (DMEs). NF-κB activation has been associated with inflammatory conditions, oxidative stress, and crosstalk with several nuclear receptors that alter the expression and activity DMEs, thereby influencing drug metabolism [34]. However, it is unclear whether NF-κB activation is involved in the AOPPs-induced decrease in DMEs in the liver. To ascertain the mechanism, we assessed the NF-κB pathway and the associated upstream proteins, including p-IKKα/β, p-IκBα, and p-NF-κB. We found that these upstream proteins were significantly upregulated (Fig. 4). In AOPPs-treated cells transfected with a luciferase reporter plasmid, the luminescence intensity was significantly increased, indicating NF-κB pathway activation (Fig. 6A). At the same time, NF-κB protein was upregulated in the cytoplasm and nucleus, with increased NF-κB protein translocation to the nucleus (Fig. 5A–C). Furthermore, the AOPPs-induced downregulation of CYP1A2 and CYP3A4 expression was significantly ameliorated by BAY-117082 and PDTC. Thus, we showed that the inhibition of the NF-κB pathway prevented the decrease in the CYP1A2 and CYP3A4 expression. Research on the precise mechanism of AOPPs-induced downregulation of DMEs is ongoing.

We also evaluated whether inflammatory cytokines can directly influence the CYP1A2 and CYP3A4 expression and activity. AOPPs are a key factor in inflammatory diseases and can induce proinflammatory cytokine release [16, 35]. The levels of multiple cytokines are increased in the plasma of patients with CKD [36]. IL-6 and TNF-α influence DME expression in vitro and in vivo [37]. In our rat model, AOPPs increased the IL-6 and TNF-α concentrations in the plasma (Fig. 7A), and they dose-dependently increased the IL-6 and TNF-α release in the rat macrophages (Fig. 7B). This result shows that AOPPs can upregulate the levels of IL-6 and TNF-α, which is consistent with previous findings. After treatment with various concentrations of IL-6 or TNF-α, the protein expression levels of CYP1A2 and CYP3A4 in HepG2 and L-02 (Fig. 7C, D) cells were dose-dependently downregulated. This result indirectly suggests that IL-6 and TNF-α may participate in the AOPPs-related regulation of CYP1A2 and CYP3A4. However, the effects of IL-6 and TNF-α occur concurrently in vivo and our IL-6 and TNF-α experiments were carried out separately in the in vitro models. Thus, additional experiments are needed to determine these specific interactions.

**Fig. 8:**
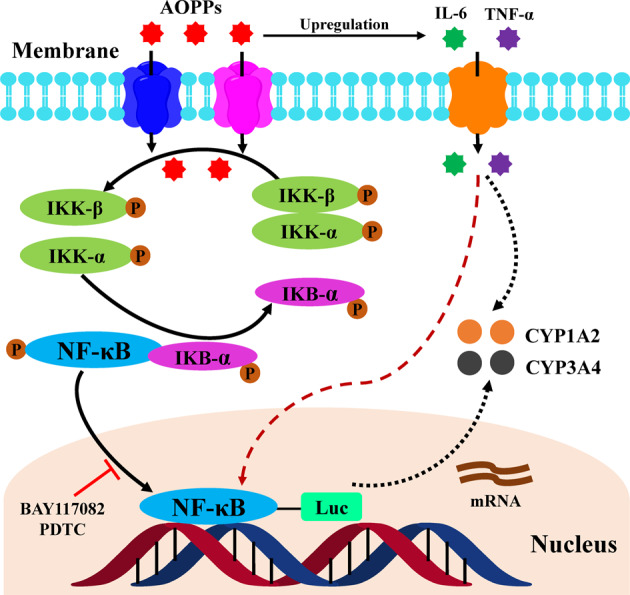
Possible mechanism underlying the AOPPs-induced CYP1A2 and CYP3A4 downregulation. This study demonstrates that AOPPs significantly decrease the CYP1A2 and CYP3A4 expression and activities via direct activation of the NF-κB pathway and induction of nuclear translocation of NF-κB to inhibit the transcription and translation of CYP1A2 and CYP3A4. As inflammatory cytokines, IL-6 and/or TNF-α may mediate this process.

This study demonstrates that AOPPs significantly decrease the expression and activities of the CYP1A2 and CYP3A4 enzymes via the NF-κB signaling pathway, and IL-6 and/or TNF-α may mediate this process (Fig. 8). To the best of our knowledge, this is the first study to systematically evaluate the effect of AOPPs on hepatic drug metabolism using rat and cellular models. These results suggest that the metabolism and elimination of drugs that are metabolized by CYP1A2 or CYP3A4 may be altered by an increase in AOPPs, and additional studies are required to determine the relationships between AOPPs and DMEs. Prospective clinical studies are needed to investigate the extent of the contribution of AOPPs to the pharmacokinetic changes related to various drugs.

## Data Availability

All data and models during the study appear in the submitted article. The online version of this article (10.1038/s41374-021-00610-9) contains supplementary material, which is available to authorized users.
